# Modified Spruce Sawdust for Sorption of Hexavalent Chromium in Batch Systems and Fixed-Bed Columns

**DOI:** 10.3390/molecules25215156

**Published:** 2020-11-05

**Authors:** Dororthea Politi, Dimitrios Sidiras

**Affiliations:** Laboratory of Simulation of Industrial Processes, Department of Industrial Management and Technology, School of Maritime and Industrial Studies, University of Piraeus, 80 Karaoli & Dimitriou, GR 18534 Piraeus, Greece; doritapoliti@yahoo.gr

**Keywords:** adsorption, hexavalent chromium, lignocellulosic biomass, pre-treatment

## Abstract

This study investigated the potential use of spruce sawdust that was pretreated with diethylene glycol and sulfuric acid for the removal of hexavalent chromium from wastewater. The sawdust pretreatment process was conducted at different temperatures and times. The adsorbent was characterized by quantitative saccharification, scanning electron microscopy, and Brunauer–Emmet–Teller surface area analysis. Adsorption capacity was studied for both batch and column processes. The experimental adsorption isotherms were simulated using seven isotherm models, including Freundlich and Langmuir models. By using the Langmuir isotherm model, the maximal Cr(VI) adsorption capacity of organosolv-pretreated spruce sawdust (*q_m_*) was 318.3 mg g^−1^. Furthermore, the kinetic data were fitted to Lagergren, pseudo-second-order, and intraparticle diffusion models, revealing that the adsorption of Cr(VI) onto spruce sawdust pretreated with diethylene glycol and sulfuric acid is best represented by the pseudo-second-order kinetic model. Three kinetic models, namely, the Bohart–Adams model, Thomas model, and modified dose–response (MDR) model, were used to fit the experimental data obtained from the column experiments and to resolve the characteristic parameters. The Thomas adsorption column capacity of the sawdust was increased from 2.44 to 31.1 mg g^−1^ upon pretreatment, thus, demonstrating that organosolv treatment enhances the adsorption capability of the material.

## 1. Introduction

The increased magnitude of industrial processes has led to overwhelming environmental pollution and enormous damage to specific ecosystems. Specifically, the high toxicity of heavy metals makes them significant environmental and public health threats. Heavy metals speak to a significant issue for the environment and for all the life forms, since they are not biodegradable and can gather in living tissues, reaching human organisms as well through the chain food [1]. The use of chromium is particularly worrying because it is being increasingly utilized in developing countries in a number of industries, such as leather tanning, electroplating, textile dyeing, and metal finishing [2].

The International Agency for Research on Cancer has concluded that the sixth oxidation state of chromium, namely Cr(VI), is carcinogenic to human beings. The World Health Organization has dictated that the highest allowable level for Cr(VI) in drinking water is 0.05 mg L^−1^ [3,4]. Furthermore, a number of countries are facing issues in relation to the presence of hexavalent chromium in natural ecosystems. For instance, in Greece, the Asopos River has been considered a “processed industrial waste receiver” since 1969. It was recently discovered that the Asopos and Thiva Basins are massively contaminated with Cr(VI) because of the excessive industrial activity around the area and unregulated discharge even though a Cr(VI) limit in industrial discharge is mandatory to acquire licensing [5]. Therefore, owing to its high toxicity, it is important to minimize Cr(VI) to tolerable levels prior to its discharge in aquatic environments. Cr(VI) forms several species, the relative proportions of which depend on both pH and total Cr(VI) concentration. Within the normal pH range in natural waters, Cr(VI) exists mainly as CrO_4_^−2^, HCrO^−4^, and Cr_2_O_7_^2−^ [6].

There are a number of processes used for the removal of Cr(VI) from aqueous solutions. The most commonly utilized techniques for the removal of Cr(VI) from wastewater incorporate reduction, precipitation, membrane filtration [7], biological method, ion exchange, and adsorption [8]. The initial five ordinary separation methods have numerous hindrances such as the high capital and operational cost, the creation of measurable chromium sludge, and potential production of secondary pollution bringing about high removal costs even though adsorption has been generally utilized for the removal of Cr(VI) from wastewater because of its straightforward operation, high removal efficiency, and low treatment cost [9].

Adsorption by using activated carbon has been demonstrated to be highly effective for decontaminating wastewater but is very expensive to perform [10]. Consequently, there is a great need to develop low-cost absorbents for the removal of Cr(VI) from aqueous environments [11]. Biomass provides a low-cost and renewable source of adsorbents and can be utilized in their original form, modified, or transformed to activated carbon. These waste materials have some to no financial value and often pose a disposal problem. Several naturally available biomasses, including sawdust [12], pistachio hull powder [13], Brazilian-pine fruit coat [14], orange peels and corncob [15], and *Melaleuca diosmifolia* leaf [16] have been evaluated as adsorbents for Cr(VI). Moreover, numerous modified lignocellulosic biomasses including cobalt-coated bamboo charcoal [17], walnut shells [18], nano-sized cellulose fibers obtained from rice husk [19], a lignin-based composite [20], biochar [21], formaldehyde created sawdust and sulfuric acid created sawdust [22], ammoniated rice straw [23], olive stones coated by iron-based nanoparticles [24], sulfuric acid and heat-treated oil palm fiber [25], activated carbon from longan seed [26], and lignin [27] have been examined as adsorbents for Cr(VI).

Numerous pretreatments on lignocellulosic biomass exist, such as autohydrolysis [28,29], alkaline pretreatment [30], acid hydrolysis [31,32], organosolv pretreatment [33,34], and many others. Organosolv pretreatment is considered promising, along with many different techniques, because of its unique potential benefits, such as separation of high purity cellulose, production of high quality lignin, higher efficiency of hemicellulose fractionation in contrast with customary treatments and organic solvent recovery [33,34].

In this study, we evaluated the efficiency of organosolv-pretreated spruce sawdust as a biosorbent for the removal of Cr(VI) from aqueous solutions. Different biosorbents were prepared by pretreating spruce sawdust with diethylene glycol and sulfuric acid at four different temperatures (160 °C, 180 °C, 200 °C, and 220 °C) and two different isothermal hydrolysis times (0 and 50 min [preheating period not included]). The physicochemical and sorption properties of the pretreated and untreated spruce sawdust were assessed by quantitative saccharification, scanning electron microscopy (SEM), and Brunauer–Emmet–Teller (BET) surface area analysis. The possible adsorption mechanism for Cr(VI) was also investigated.

## 2. Results and Discussion

### 2.1. Organosolv Pretreatment

Table 1 displays the results of pretreatment with 50% diethylene glycol/50% H_2_O and pretreatment with 50% diethylene glycol/50% H_2_O/0.045 N H_2_SO_4_. The percentage of lignin in the materials increases with the intensity of the pretreatment. By changing the isothermal pretreatment time, the percentage of lignin in the materials increases.

For the pretreatment with 50% diethylene glycol/50% H_2_O, there is a relative stability for the percentage of cellulose at 200 °C and 220 °C for an isothermal reaction time of 50 min. Additionally, for pretreatment with 50% diethylene glycol/50% H_2_O/0.045 N H_2_SO_4_, the percentage of cellulose following isothermal reaction for 50 min at 220 °C is low.

Table 1 shows that, for pretreatment with 50% diethylene glycol/50% H_2_O, more extreme pretreatment conditions lead to smaller percentages of xylan and mannan. Conversely, for pretreatment with 50% diethylene glycol/50% H_2_O/0.045 N H_2_SO_4_, a steep decrease in mannan is observed, particularly for an isothermal reaction time of 0 min. 

The solid residue yield (SRY) decreases with pretreatment temperature for both systems. The temperatures used were 160 °C, 180 °C, 200 °C, and 220 °C, which were reached after 42, 50, 62, and 80 min preheating, respectively. The SRY is lower for an isothermal reaction time of 50 min in relation to an isothermal reaction time of 0 min.

### 2.2. BET Surface Area

Table 2 displays the specific surface areas of the materials for all pretreatment conditions. There is an increase in the specific surface area upon increasing the pretreatment temperature. For the pretreatment with 50% diethylene glycol/50% H_2_O, the largest specific surface area appears to be 4.078 m^2^g^−1^, which is achieved by pretreatment at 220 °C for 50 min. For the pretreatment with 50% diethylene glycol/50% H_2_O/0.045 N H_2_SO_4_, the largest specific surface area appears to be 11.335 m^2^g^−1^, which was achieved by pretreatment at 220 °C for 50 min.

### 2.3. Microstructure

**Figure 1:**
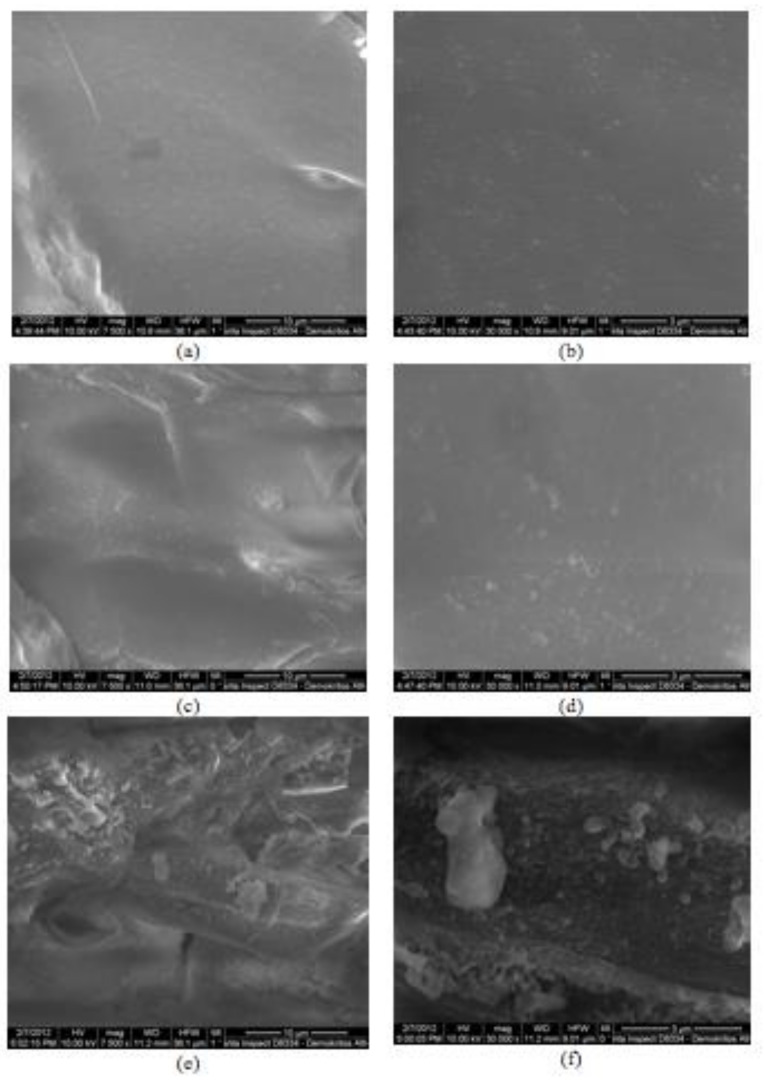
SEM micrographs for untreated, (**a**,**b**), diethylene glycol (180 °C, 50 min) pretreated, (**c**,**d**) and diethylene glycol/0.045 N H_2_SO_4_ (180 °C, 50 min) pretreated spruce, (**e**,**f**). Magnification (**a**,**c**,**e**) ×7500 and (**b**,**d**,**f**) ×30,000.

Figure 1 shows the SEM micrographs of untreated spruce sawdust and those treated under acid-catalyzed organosolv conditions at 180 °C for 50 min at two different magnifications (×7500 and ×30,000). The surface of the pretreated material exhibits greater roughness than the untreated material. This is particularly evident at ×30,000 magnification.

### 2.4. Adsorption Isotherms

**Figure 2:**
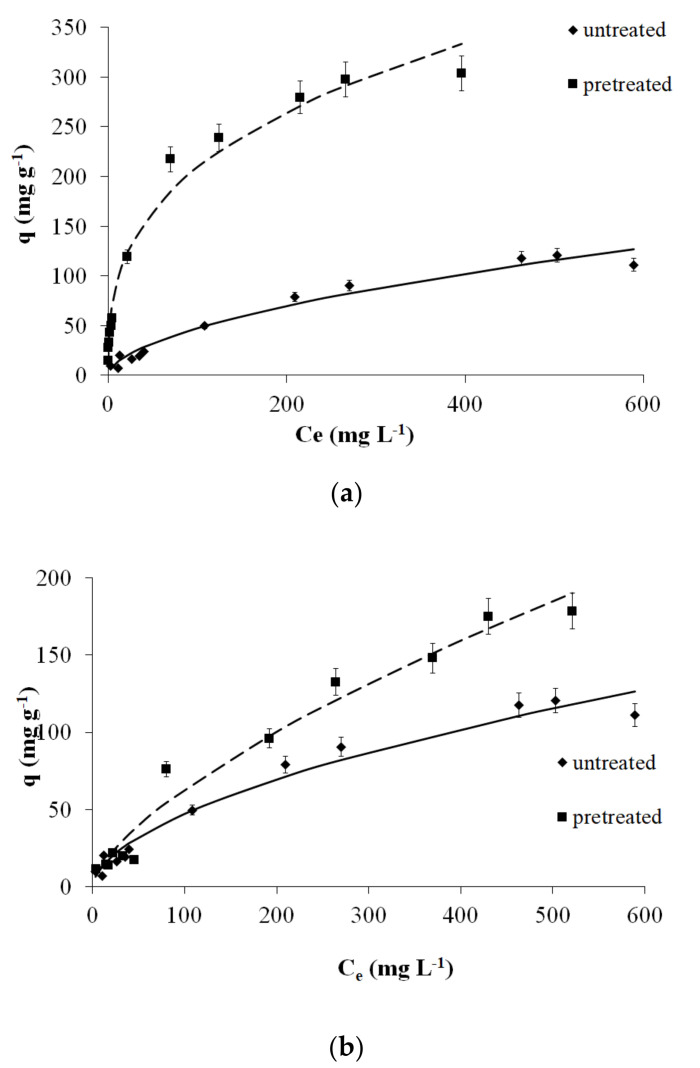
The Freundlich isotherms of Cr(VI) adsorption on untreated and pretreated (**a**) with diethylene glycol/sulfuric acid (180 °C, 50 min) and (**b**) with diethylene glycol (220 °C, 50 min) spruce sawdust.

Therefore, in this study, seven isotherm models were applied to fit the experimental results (Figure 2). The Freundlich [35] isotherm is given by the following equation.

(1) $$q=K_{F}\cdot {\left(C_{e}\right)}^{\frac{1}{n}}$$

where *q* is the amount adsorbed per unit mass of the adsorbent (mg g^−1^), *C_e_* is the equilibrium concentration of the adsorbate (mg L^−1^), and *K_F_*, *n* is the Freundlich constants related to adsorption capacity and intensity, respectively. Equation (1) in logarithmic form gives the following.

(2) $$logq=logK_{F}+\frac{1}{n}logC_{e}$$

The Langmuir isotherm [36] is given by the following equation.

(3) $$q=\frac{K_{L}q_{m}C_{e}}{1+K_{L}C_{e}}$$

or

(4) $$\frac{1}{q}=\left(\frac{1}{q_{m}}\right)+\left(\frac{1}{K_{L}\cdot q_{m}}\right)\cdot \left(\frac{1}{C_{e}}\right)$$

where *K_L_* is the Langmuir constant related to the energy of adsorption (L mg^−1^) and *q_m_* is the amount of Cr(VI) adsorbed (mg g^−1^) when saturation is attained. The parameters *K_L_* and *q_m_* can be obtained either by plotting 1/*q* versus 1/*C_e_* or by non-linear regression analysis. The characteristics of the Langmuir isotherm can be described by a dimensionless constant called the ‘equilibrium parameter’ or ‘separation factor’ *R_L_*.

(5) $$R_{L}=\frac{1}{1+K_{L}\cdot C_{0}}$$

where *C*_0_ is the initial concentration (mg L^−1^) and *K_L_* is the Langmuir constant (L mg^−1^). The value of *R_L_* indicates whether the type of the proposed isotherm is either unfavorable (*R_L_* > 1), linear (*R_L_* = 1), favorable (0 < *R_L_* < 1), or irreversible (*R_L_* = 0).

The Sips (Langmuir-Freundlich) [37] isotherm equation is shown below.

(6) $$q=\frac{q_{m}\cdot {\left(K_{L}\cdot C_{e}\right)}^{1/n}}{1+{\left(K_{L}\cdot C_{e}\right)}^{1/n}}\;\mathrm{or}\;\frac{1}{q}=\left(\frac{1}{q_{m}}\right)+\left(\frac{1}{K_{L}^{1/n}\cdot q_{m}}\right)\cdot {\left(\frac{1}{C_{e}}\right)}^{1/n}$$

where *K_L_* and *q_m_* are the Langmuir constants, and *n* is the Freundlich constant.

The Radke-Prausnitz [38,39] isotherm equation is shown below.

(7) $$q=\frac{K_{L}\cdot q_{m}\cdot C_{e}}{1+K_{L}\cdot C_{e}{}^{1/n}}$$

The Modified Radke-Prausnitz [39] isotherm equation is below.

(8) $$q=\frac{K_{L}\cdot q_{m}\cdot C_{e}}{{\left(1+K_{L}\cdot C_{e}\right)}^{1/n}}$$

The Tóth [40] isotherm equation is below.

(9) $$q=\frac{q_{m}\cdot C_{e}}{{\left(1/K_{L}+C_{e}{}^{n}\right)}^{1/n}}$$

The Unilan [39] isotherm equation is shown below.

(10) $$q=\frac{q_{m}}{2s}\ln\left(\frac{1+K_{L\cdot }C_{e}\cdot e^{s}}{1+K_{L}\cdot C_{e}\cdot e^{-s}}\right)$$

where *s* is a new constant.

Table 3 presents all the parameters of the Freundlich and Langmuir models using nonlinear regression analysis (NLRA) for all the samples with different pretreatment conditions (temperature, isothermal treatment time, and added acid). The standard error of estimate (SEE) was calculated in each case by the following expression.

(11) $$SEE=\sqrt{\overset{n^{\prime }}{\underset{i=1}{\sum }}\frac{{\left(y_{i}-y_{i,theor}\right)}^{2}}{n^{\prime }-p\prime }}$$

where *y_i_* is the experimental value of the depended variable, *y_i,theor_* is the theoretical or estimated value of the depended variable, *n′* is the number of experimental measurements, and *p′* is the number of parameters (the difference [*n’* − *p′*] being the number of degrees of freedom). The fitting of the Freundlich adsorption model to the experimental data is very satisfactory (see Table 3 and Figure 2a,b).

Figure 2a,b shows the Freundlich isotherms for Cr(VI) adsorption on untreated spruce sawdust and those pretreated with (a) 50% diethylene glycol/50% H_2_O/0.045 N H_2_SO_4_ at 180 °C for 50 min and (b) 50% diethylene glycol/50% H_2_O at 220 °C for 50 min.

Table 3 shows the Freundlich isotherm capacity coefficient *K_F_* of Cr(VI) adsorption on untreated and pretreated spruce sawdust. According to the same table with the *K_F_* measurement for Cr(VI) removal, it can be observed that pretreatment at 160 °C provides insufficient improvements to the adsorption capacity of the material. By contrast, more intense pretreatment, such as pretreatment at 220 °C for 0 min and pretreatment at 180 °C for 50 min, yield significant improvements in *K_F_* adsorption capacity.

In relation to industrial applications, the adsorption capacity *q_m_* is given in Table 3 according to the Langmuir model. On this basis, pretreatment with an organic solvent catalyzed by acid significantly improves *q_m_*. Pretreatment at 160 °C does not improve the adsorption capacity of our material for time 0. By contrast, for the more intense pretreatment temperatures of 180 °C, 200 °C, and 220 °C, we see a significant improvement with a maximum temperature of 220 °C for 0 min (*q_m_* = 257.2 mg g^−1^). Conversely, for pretreatment with organic solvent catalyzed by acid for an isothermal time of 50 min, improvement is observed for the two lowest pretreatment temperatures (160 °C and 180 °C) with maximum temperatures of 180 °C for 50 min, as shown in Table 3, where *q_m_* = 318.3 mg g^−1^ for the Langmuir model.

In the current study, the *R_L_* values were calculated in the range of zero to one for all initial Cr(VI) concentrations (*C*_0_) and for all adsorbents considered. This indicates a sympathetic adsorption. By contrast, *R_L_* > 1 represents an adverse adsorption, and *R_L_* = 1 represents a linear adsorption. Furthermore, the adsorption is permanent if *R_L_* = 0. 

Table 4 presents the parameters of seven isothermal models in their nonlinear form, which is similar to previous pretreatments. The materials for which the parameters of the seven isothermal models are presented are untreated spruce sawdust, sawdust pretreated with 50% diethylene glycol/50% H_2_O at 220 °C for 50 min, and sawdust pretreated with 50% diethylene glycol/50% H_2_O/0.045 N H_2_SO_4_ at 180 °C for 50 min, which has the optimum adsorption capability. The fitting of the Sips model was better when compared to other six isotherms models but also Freundlich and Langmuir models were very satisfactory using only two parameters.

In Table 5, we compare the Cr(VI) adsorption parameters *K_F_*, *n*, *q_m_*, and *K_L_* for pretreated and untreated spruce sawdust with other adsorbents derived from agricultural or waste materials, according to available literature data. The optimal *q_m_* value found in this work was higher than the corresponding values of most of the other materials in Table 5, while the *K_F_* was the highest.

### 2.5. Adsorption Kinetics

Various kinetic models have been used to identify the reasonable mechanisms for solid/liquid adsorption frameworks. Among them, Lagergren’s pseudo-first-order [47] and pseudo-second-order kinetic models [48] as well as the intraparticle diffusion kinetic model [49] are the three most common models. Thus, these three kinetic models were utilized to fit the experimental data for the adsorption of Cr(VI) on pretreated spruce sawdust.

The widely used Lagergren equation [47] or the pseudo-first order kinetic model is shown below.

(12) $$q-q_{t}=q\cdot e^{-k\cdot t}$$

where *q* and *q_t_* are the amounts of Cr(VI) adsorbed per unit mass of the adsorbent (in mg g^−1^) at equilibrium time (t→∞) and adsorption time *t*, respectively, while *k* is the pseudo-first order rate constant for the adsorption process (in min^−1^).

(13) $$q=\left(C_{0}-C_{e}\right)V/m\;\mathrm{and}\;q_{t}=\left(C_{0}-C\right)V/m$$

where *C*, *C*_0_, and *C_e_* are the concentrations of Cr(VI) in the bulk solution at time *t*, 0, and ∞, respectively, while *m* is the weight of the adsorbent used (in g), and *V* is the solution volume (in mL). Further modification of Equation (12) in logarithmic form gives the following.

(14) $$\ln(q-q_{t})=\ln q-k\cdot t$$

The pseudo second order kinetic model [48] is as follows.

(15) $$q_{t}=q-{\left[q^{-1}+k_{2}t\right]}^{-1}\;\mathrm{or}\;q_{t}=q-\frac{1}{\frac{1}{q}+k_{2}t}$$

where *k*_2_ (min^−1^) is the rate constant of second order adsorption.

The possibility of intra-particle diffusion was explored by using the intra-particle diffusion model [49].

(16) $$q_{t}=c+k_{p}\cdot \sqrt{t}$$

where *q_t_* is the amount of Cr(VI) adsorbed at time *t*, *k_p_* (mg g^−1^ min^−0.5^) is the intra-particle diffusion rate constant, and *c* (mg g^−1^) is a constant related to the thickness of boundary. A value of c close to zero indicates that diffusion is the only controlling step of the adsorption process.

The most appropriate model was chosen in terms of both SEE and *q_e_* values. Table 6 and Table 7 show the kinetic results.

**Figure 3:**
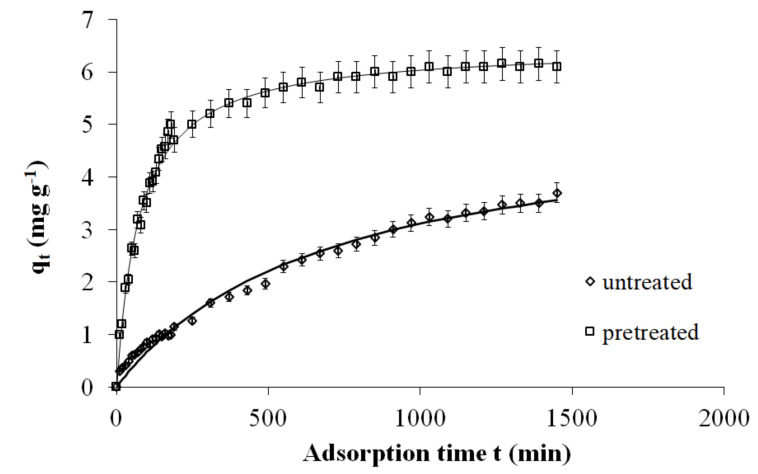
The second-order kinetics of Cr(VI) adsorption on untreated and pretreated with 50% diethylene glycol/50% water/0.045 N H_2_SO_4_, 180 °C, 50-min spruce sawdust. Adsorption: 23 °C, initial concentration *C*_0_ = 7 mg L^−1^ for Cr(VI), m/V = 1 g L^−1^ pH = 2.

Figure 3 shows the second-order kinetics of Cr(VI) adsorption on untreated and pretreated spruce sawdust. Pretreatment with 50% diethylene glycol/50% H_2_O/0.045 N H_2_SO_4_ at a medium temperature (180 °C) leads to the highest adsorbance.

The SEE values for the second-order kinetic models are 0.133 for untreated spruce sawdust and 0.150 for spruce sawdust pretreated with 50% diethylene glycol/50% H_2_O/0.045 N H_2_SO_4_ at 180 °C for 50 min.

For pretreatment with 50% diethylene glycol/50% H_2_O, the NLRA estimates for *k_2_* of the second-order kinetic model return values from 0.0003–0.0008 g mg^−1^min^−1^, and the range of SEE error values is 0.079–0.181. However, for pretreatment with 50% diethylene glycol/50% H_2_O/0.045 N H_2_SO_4_, the NLRA estimates for the *k_2_* of the second-order kinetic model return values from 0.0003–0.0021 g mg^−1^min^−1^, and the range of SEE error values is 0.106–0.178. Therefore, these SEE values are lower than those of the first-order kinetic model. According to the above, the pseudo-second-order kinetic model demonstrates that the equilibrium amount of adsorbed absorbate controls the number of binding sites [50].

**Figure 4:**
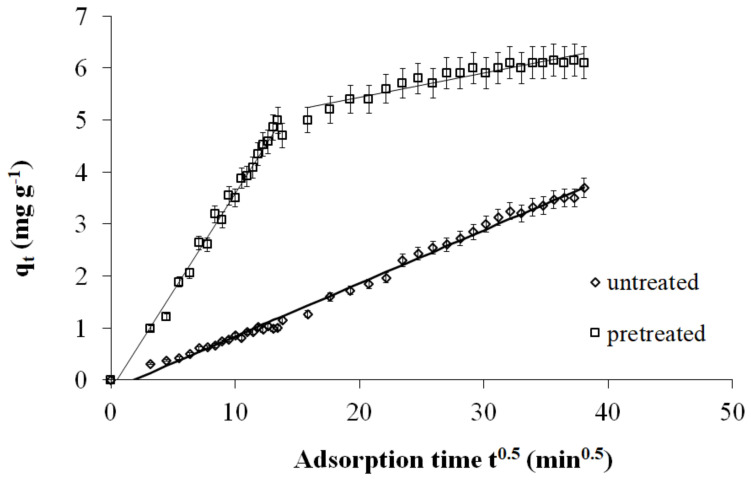
The Intraparticle kinetics of Cr(VI) adsorption on untreated and pretreated with 50% diethylene glycol/50% water/0.045 N H_2_SO_4_, 180 °C, 50 min spruce sawdust. Adsorption: 23 °C, initial concentration *C*_0_ = 7 mg L^−1^ for Cr(VI), m/V = 1 g L^−1^ pH = 2.

Finally, the NLRA estimates for the *k*_p_ of the intraparticle diffusion model for the pretreatment with 50% diethylene glycol/50% H_2_O, for 0 min, at 160 °C, 180 °C, 200 °C, and 220 °C, are 0.1133, 0.1011, 0.1203 and 0.1655, respectively. Therefore, according to the intraparticle diffusion model, a higher pretreatment temperature corresponds to higher values of *k_p_*. The SEE values of the intraparticle diffusion model are 0.122–0.203. The minimum value is for untreated spruce sawdust, and the maximum value is for spruce sawdust pretreated with 50% diethylene glycol/50% H_2_O/0.045 N H_2_SO_4_ at 180 °C for 50 min (see Figure 4). The intraparticle diffusion model involves a multistage adsorption process that includes the mass transfer of adsorbate molecules to the external surface of the adsorbent, their mass transfer to the internal surface of the adsorbent, and their sorption on the active sites of the adsorbent [51].

Assessing these three kinetics models demonstrated that the material improves substantially when it is pretreated with sulfuric acid, diethylene glycol, and water. 

All SEE error values for the second-order kinetic model were found to be somewhat lower than those of the Lagergren and intraparticle diffusion models, indicating the marginally higher suitability of second-order kinetics in the adsorption of Cr(VI) by spruce sawdust pretreated with organic solvent.

**Figure 5:**
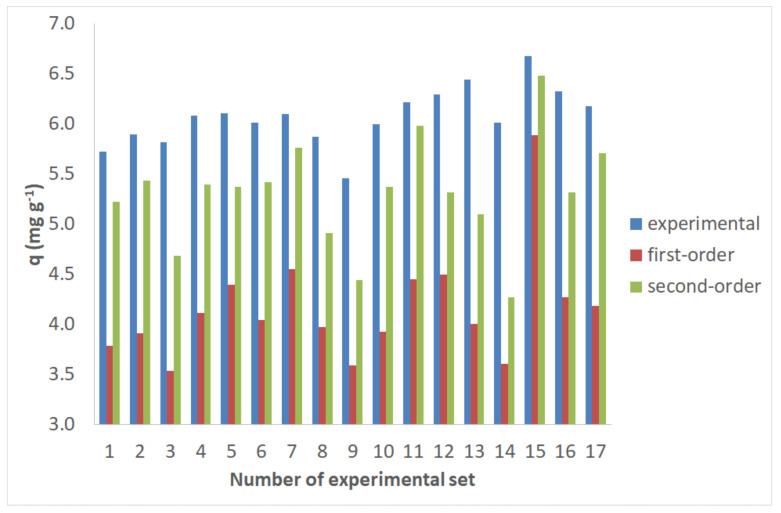
Comparison of the experimental equilibrium uptakes to the simulated *q*-values obtained via pseudo-first and pseudo-second order kinetic models.

According to the findings presented in Figure 5, the simulated *q*-values obtained via pseudo-first and pseudo-second order kinetic models were decreased by 31% and 13%, respectively (as an average), when compared to the experimental equilibrium uptakes achieved by isothermal experiments (equilibrium reached after seven days).

### 2.6. Adsorption Columns

For the adsorption column experiments, the ‘bed depth service model’ developed by Bohart and Adams [52] is commonly used as follows.

(17) $$\ln\left(\frac{C_{i}}{C}-1\right)=\frac{K\cdot N\cdot x}{u}-K\cdot C_{i}\cdot t$$

where *C* = effluent concentration (mg L^−1^), *C_i_* = influent concentration (mg L^−1^), *K* = adsorption rate coefficient (L mg^−1^ min^−1^), *N* = adsorption capacity coefficient (mg L^−1^), *x* = bed depth (cm), *u* = linear velocity (cm min^−1^), and *t* = adsorption time (min). 

The Thomas [53] model is one of the most widely used models in the column performance theory. The main difference between the Bohart-Adams and the Thomas model is the form of the sorption isotherm assumed. The latter assumes a Langmuir (favorable) isotherm. It has been shown that, when the sorption isotherm is highly favorable, the actual Thomas model reduces to the Bohart-Adams model. The expression by Thomas for an adsorption column is:

(18) $$\ln\left(\frac{C_{i}}{C}-1\right)=\frac{k_{T}q_{0}M}{Q}-k_{T}\cdot C_{i}\cdot t$$

where *k_T_* is the Thomas rate constant, *q_0_* is the sorption capacity of the adsorbent per unit mass of the adsorbent, *M* is the mass of adsorbent, and *Q* is the flow rate. It is evident that Equations (17) and (18) look strikingly similar even at first glance. They are mathematically identical and have interchangeable parameters [54,55].

The Modified Dose–Response (MDR) model was proposed by Yan et al. [55] because it minimizes the error resulting from the use of the Thomas model, especially at lower and higher time periods of the breakthrough curve. The MDR model is expressed as:

(19) $$\frac{C}{C_{i}}=1-\frac{1}{1+{\left[V/b_{mdr}\right]}^{a_{mdr}}}\;\mathrm{or}\;\frac{C}{C_{i}}=1-\frac{1}{1+{\left[C_{i}V/\left(q_{0}M\right)\right]}^{a_{mdr}}}$$

where *b_mdr_ = (q*_0_*M)/C_i_* and *α_mdr_* are the modified dose–response constants, and *V = Q^.^t*.

Table 8 shows the parameters of the Bohart–Adams, Thomas, and MDR models for untreated and pretreated spruce sawdust for Cr(VI) removal. The pretreatment conditions of spruce sawdust are 50% diethylene glycol/50% H_2_O/0.045 N H_2_SO_4_ for 160 °C, 180 °C, 200 °C, and 220 °C, and the isothermal reaction time is 50 min. The experiments were performed at a flow rate of 2 mL min^−1^, the mass of the material was 18 g, and the Cr(VI) concentration was 60 mg L^−1^.

According to Table 8, the Bohar-Adams capacity *N* (mg L^−1^) for the untreated spruce sawdust is 662 mg L^−1^ for the material pretreated with 50% diethylene glycol/50% H_2_O/0.045 N H_2_SO_4_ at 180 °C for 50 min is 7814 mg L^−1^, which is the maximum value of *N*. However, for the most severe pretreatment conditions (50% diethylene glycol/50% H_2_O/0.045 N H_2_SO_4_ at 220 °C for 50 min), the value of *N* decreases at 998 mg L^−1^.

Furthermore, we observe that, according to the Thomas model, the adsorption capacity *q*_0_ (mg g^−1^) for the untreated material is equal to 2.437 mg g^−1^, while that for the material pretreated at 180 °C for 50 min is 31.08 mg g^−1^. Consequently, the pretreated material at the optimal conditions has an adsorption capacity that is 13 times that of the untreated material. However, for the most severe pretreatment conditions, it is reduced to 4.452 mg g^−1^.

Similarly, the MRD model shows that the material pretreated at 180 °C for an isothermal reaction time of 50 min has an adsorption capacity that is 14 times that of the untreated material. For the extreme pretreatment at 220 °C for 50 min, the adsorption capacity of the material is greater than that of the unprocessed material but was not optimal, as we expected. In general, the adsorption capacity *q*_0_ (mg g^−1^) values estimated, according to the MDR model, are very similar to those of the Thomas model, resulting in the same conclusion with regard to the process optimization.

**Figure 6:**
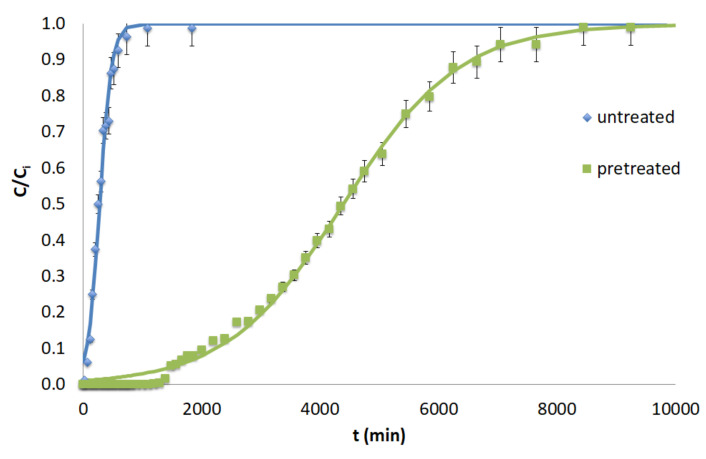
Breakthrough curves of Cr(VI) adsorption, *C*/*C_i_* versus t (min) according to the Thomas model, on untreated and pretreated (with 50% diethylene glycol/50% water/0.045 N H_2_SO_4_, 180 °C, 50 min) spruce sawdust. Adsorption: *Q* = 2 mL min^−1^, initial concentration *C*_0_ = 60 mg L^−1^ for Cr(VI), M = 18 g, pH = 2.

Figure 6 shows the theoretical curves from the Thomas model for untreated and pretreated with the acid-catalyzed organic solvent spruce. The fitting of the Thomas (or the Bohart–Adams) model to the experimental data is not always better than that of the MDR model but it fits better than the high *C/C_i_* experimental values while the MDR model fits the very low initial *C/C_i_* values better.

The Cr(VI) adsorption capacity according to the Thomas capacity parameter *q*_0_ (mg L^−1^) of organosolv-pretreated spruce sawdust was compared with those of other adsorbents reported in the literature (Table 9). According to the Thomas model, spruce sawdust pretreated with 50% diethylene glycol/50% H_2_O/0.045 N H_2_SO_4_ at 180 °C for an isothermal reaction time of 50 min was *q*_0_ = 31.08 mg g^−1^. The Cr(VI) adsorption capacity for the organosolv-pretreated spruce sawdust is comparable than the reported values of some previously studied adsorbents. Therefore, it can be concluded that organosolv-pretreated spruce sawdust is competitive to other modified adsorbents lignocellulosic adsorbents.

### 2.7. Chemical-Physical Mechanisms of the Process

Adsorption on sawdust can be physical, ion exchange, and chemical [12]. Generally, three types of mechanisms are involved in an adsorption process: (i) film diffusion, which involves the movement of adsorbate molecules from the bulk of the solution toward the external surface of the adsorbent, (ii) particle diffusion, where the adsorbate molecules move in the interior of the adsorbent particles, and (iii) adsorption of the adsorbate molecules on the interior of the porous adsorbent [61,62]. 

The Thomas kinetic model satisfactorily described the column adsorption. This model is based on the mass transfer model, which postulates that adsorbate emigrates from the solution to a film around the particle and expands through the liquid film to the surface of the adsorbent. Subsequently, this step is followed by intraparticle diffusion and adsorption on the active site, assuming Langmuir isotherm for equilibrium, plug flow performance in the bed, and second-order reaction kinetics [12,63]. 

Moreover, to predict the rate determining the diffusion mechanism within the biosorption system above, applied an intraparticle diffusion model that proved to be very effective. Webber’s pore diffusion model was applied on the kinetic data with the pore diffusion factor described by Equation (16), where *k_p_* is the intra-particle diffusion rate constant. In the case that intraparticle diffusion is the rate limiting step, the *q* versus *t*^0.5^ will be linear with slope *k_p_* and the plot will pass through the origin, i.e., intercept *c* = 0. Otherwise, some other mechanism along with intraparticle diffusion is involved in the biosorption process, such as film diffusion. Based on the kinetics constants presented in Table 7 and the interpretation of the pretreated sawdust data in Figure 4, the sorption process consists of two steps. The linearization did not pass through the origin, indicating that intraparticle diffusion is not the rate-limiting step and implying that the biosorption is affected by more than one process. Additionally, in the same figure, the first part refers to the film diffusion and the second part correlates with the diffusion within the adsorbent. The initial segment in the plot specified an external mass transfer while the second segment is caused by intraparticle diffusion or pore diffusion [64,65]. With regard to the untreated sawdust, the second part does not appear within 24 h of our experiment (see Figure 4).

According to the literature, Cr(VI) can be adsorbed either by cellulose (modified or not, activated or not) [19,66] either by lignin (obtained from lignocellulosic biomass in combination with some modification and/or activation) [20,27,67,68]. According to the present experiments, there are optimal conditions for achieving maximum Cr(IV) adsorption capacity by material containing both cellulose and lignin. According to the presented data, there is no need to achieve maximum cellulose concentration or maximum lignin concentration (Table 1). Even the BET surface area (Table 2), maximization is not necessary or desired to obtain optimized adsorption material.

Studying the mechanism of Cr(VI) biosorption on the untreated and pretreated sawdust surface is important for understanding the enhanced removal of Cr(VI) from aqueous solution using pretreated sawdust. FTIR was used as a qualitative technique for assessing the chemical structure of sawdust [65].

**Figure 7:**
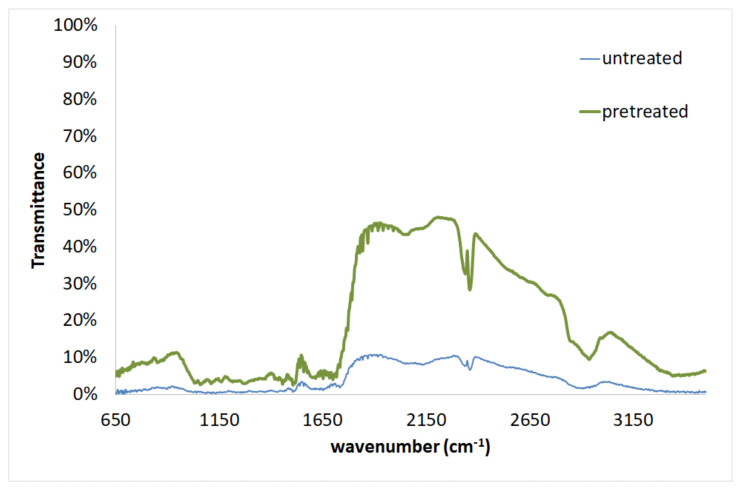
FTIR for untreated and pretreated spruce sawdust.

The FTIR spectrum of the untreated spruce and of the pretreated (50% diethylene glycol/50% water/0.045 N H_2_SO_4_, 180 °C, 50 min) spruce sawdust is provided in Figure 7. The comparison of these FTIR spectrums shows that some peaks were shifted. Moreover, the transmittance was significantly increased with regard to the pretreated sawdust compared to the untreated material. The major peaks, the shift of the peaks, the assignment, and the corresponding component are presented in Table 10. In the case of the pretreated material, there is a peak shift at wavenumber 3347 cm^−1^ (decreased by 117 cm^−1^ compared to the peak at 3464 cm^−1^ for the untreated sawdust) representing the O-H stretching existing in cellulose, hemicelluloses, and lignin. Moreover, a peak shift at 2942 cm^−1^ (increased by 33 cm^−1^ compared to the 2909 cm^−1^ for the untreated material) indicates the presence of C-H stretching and a peak shift at 1700 cm^−1^ (decreased by 35 cm^−1^ compared to the 1735 cm^−1^ for untreated) indicates the C=O stretching. In addition, a peak shift at 1457 cm^−1^ (increased by 22 cm^−1^ compared to the 1435 cm^−1^ for the untreated) can be assigned to C-H deformation existing in lignin. The peak shift at 1043 cm^−1^ (increased by 25 cm^−1^ compared to the 1068cm^−1^ for the untreated) can be attributed to C-OH stretching vibration and C-O deformation. The appearance of a new peak at 1039 cm^−1^ can be attributed to the C-O stretching and aromatic C-H in plane deformation. Finally, the peak shifts at 855 and 851 cm^−1^ in the FTIR spectrum of pretreated spruce sawdust may be due to the C-O-C stretching and aromatic C-H out of plane bending, respectively. These peaks exist in cellulose/hemicelluloses and in lignin, respectively.

**Figure 8:**
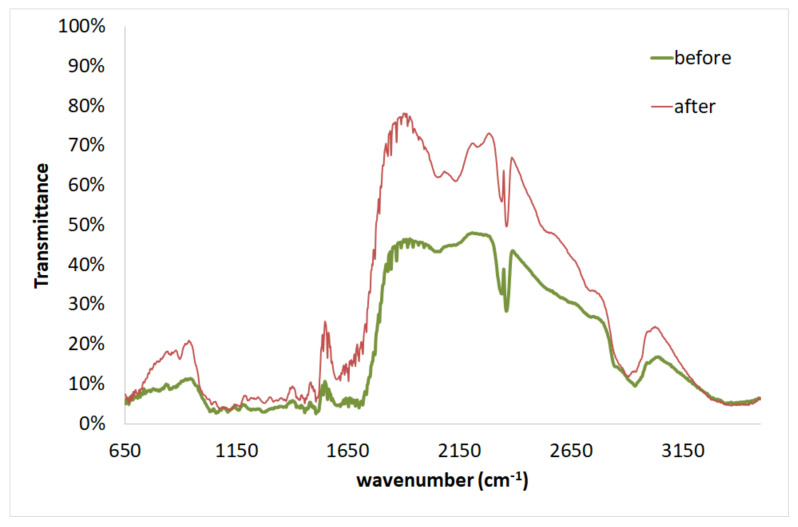
FTIR for pretreated spruce sawdust before and after Cr(VI) adsorption.

In Figure 8, the transmittance of the FTIR spectra of the pretreated (50% diethylene glycol/50% water/0.045 N H_2_SO_4_, 180 °C, 50 min) spruce sawdust before and after Cr(VI) adsorption are given. The adsorption conditions were *C*_0_ = 7 mg L^−1^, *t* = 1450 min, and adsorbent = 1 g L^−1^. The Transmittance was significantly increased after Cr(VI) adsorption on pretreated sawdust. The major peaks are given in Table 11. There is a peak shift at a wavenumber of 3410 cm^−1^ (increased by 63 cm^−1^ compared to the 3347 cm^−1^ before adsorption) representing the O-H stretching existing in cellulose, hemicelluloses, and lignin. Furthermore, a peak shift at 1684 cm^−1^ (decreased by 16 cm^−1^ compared to the 1700 cm^−1^ before adsorption) indicates the C=O stretching. In addition, a peak shift at 1076 cm^−1^ (increased by 8 cm^−1^ compared to the 1068 cm^−1^ before adsorption) can be attributed to the C-OH stretching vibration and C-O deformation. Finally, the peak shift at 850 cm^−1^ in the FTIR spectrum of pretreated spruce sawdust after Cr(VI) adsorption may be due to the C-O-C stretching. Shifts or changes of these peaks would indicate interactions between the Cr(VI) with functional groups on the solid surface as a result of biosorption or a chemical reaction. Consequently, the increases in the transmittance of these peaks indicated the oxidation of cellulose/lignin in the occurrence of the adsorbed Cr(VI). In addition, it can be seen that the whole region of different functional group bands on the sawdust surface were involved in the biosorption of Cr(VI).

The FTIR spectrum confirms the changes in the functional groups and surface properties of spruce sawdust, as shown by the shift of some functional groups. Table 10 and Table 11 show a number of major Transmittance peaks indicating the characteristic structure of the lignocellulosic complex before and after biosorption of Cr(VI), which is in agreement with those reported in the literature [65,69,70,71,72,73].

## 3. Materials and Methods

### 3.1. Materials

As an example, the source for industrial applications, which was a local furniture construction company, was used to acquire the spruce sawdust. At the point when the material was obtained, the moisture content was 8.3% (*w*/*w*). The portion with particle sizes of 0.2–1 mm was isolated. Table 1 shows the contents of the raw material.

### 3.2. Organosolv Pretreatment

The organosolv pretreatment processes were performed in a 3.75 L batch reactor (PARR 4843, Parr Instrument Company, Moline, IL, USA). The isothermal treatment time was 0 or 50 min (excluding the nonisothermal preheating and the cooling periods). The reaction was either (i) autocatalyzed (i.e., catalyzed by the organic acids generated by the spruce sawdust itself due to autohydrolysis during treatment) or (ii) catalyzed by 0.045 N H_2_SO_4_ at a liquid-solid ratio of 10:1. The liquid phase volume was 1000 mL diethylene glycol and 1000 mL water. The spruce sawdust loading was 200 g, and the stirring speed was 150 rpm. The temperatures used were 160 °C, 180 °C, 200 °C, and 220 °C, which were reached after the preheating periods of 42, 50, 62, and 80 min, respectively.

### 3.3. Characterization

For the quantitative saccharification of the untreated spruce sawdust and the organosolv reaction solid residues, the technique of Saeman et al. (1945) [74] was used. High-performance liquid chromatography (HPLC) (Agilent 1200, Agilent Technologies, Santa Clara, CA, USA) with an Aminex HPX-87H Column as well as a refractive index detector and 5 mM H_2_SO_4_ in water as the mobile phase was utilized for the glucose, xylose, and arabinose analyses of the filtrates from the quantitative saccharification. Cellulose was evaluated as glucan, and Hemicellulosess were assessed as xylan and arabinan [75]. Lastly, according to the Tappi T222 om-88 method (Tappi 1997) [76], the acid-insoluble lignin (Klason lignin) was established.

BET theory rationalizes the physical adsorption of gas molecules on a solid surface and serves as a basis for measuring the specific surface area of a material. Our measurements were made according to DIN 66,132 (DIN 66132, 1975) [77] by using liquid nitrogen (N_2_) with a Nova^®^ Surface Area Analyzer (Quantachrome Instruments, Boynton Beach, FL, USA) for approximately 20 h at 105 °C.

The SEM apparatus used was a JEOL JSM-6700F field-emission scanning electron microscope (JEOL Ltd., Tokyo, Japan). The magnifications used were ×7500 and ×30,000.

Fourier transform infrared (FTIR) spectra were obtained using a spectroscope (MAGNA-IR 750 Spectrometer, Serrie II, Nicolet, Madison, WI, USA). The sampling technique used herein was diffuse reflectance. The powder samples were scanned for a wavenumber of 650–3500 cm^−1^.

Hexavalent chromium was monitored by the 1,5-diphenylcarbohydrazide strategy by utilizing solitary dry powder detailing. This reagent contains an acidic buffer joined with 1,5-diphenylcarbohydrazide, which provides a purple color when hexavalent chromium is present. The test outcomes were measured at 540 nm by using a HACH DR4000U UV-visible spectrophotometer (Loveland, CO, USA), i.e., HACH Method 8023. The highest concentration that can be estimated with this technique is 0.7 mg L^−1^. Thus, we used a dilution factor of 10–1000. Each sample was measured in triplicate to eliminate dilution error.

pH measurements were performed using a WTW MultiLab model 540 (Weilheim, Germany) computerized pH meter.

### 3.4. Adsorption Isotherm Studies

Adsorption isotherms were obtained from batch tests conducted in 500 mL Erlenmeyer flask using a JULABO’s digital SW22 Shaking Water Bath (JULABO GmbH, Seelbach, Germany). The sorbent weight was 2 g (i.e., m/V = 4 g L^−1^), the temperature was 23 °C, and the initial Cr(VI) concentration varied from 15 to 700 mg L^−1^. K_2_Cr_2_O_7_ was used as the Cr(VI) source.

The pH of the solutions was set to two by utilizing diluted H_2_SO_4_. The jugs were fixed and mechanically tumbled for seven days. This timeframe was chosen following the initial investigations to accomplish the equilibrium conditions.

The utilization of adsorption isotherms is extremely helpful in investigating the interaction between the adsorbate and adsorbent in any framework. The parameters received from diverse models provide vital information on the surface properties and affinities of the adsorbent. There are several conditions for breaking down exploratory adsorption equilibrium information, and the best known surface adsorption models for single solute frameworks are the Langmuir and Freundlich models.

### 3.5. Kinetic Adsorption Studies

Batch adsorption rate tests were performed in a 4-L totally blended glass reactor fitted with a bent cutting-edge compost stirrer working at 600 rpm to maintain the lignocellulosic material in suspension. The sorbent weight was 4 g (i.e., m/V = 1 g L^−1^), and the underlying Cr(VI) fixation was 7 mg L^−1^. The effect of contact time on the uptake of Cr(VI) was evaluated in batch tests. Furthermore, the pH was adjusted equal to 2 (the pH of the solutions was balanced utilizing diluted H_2_SO_4_).

### 3.6. Adsorption Column Studies

Experiments with adsorption columns were performed on a 15 × 2.5 cm diameter column that contained 18 g of sorbent. The flow rate was set equal to 2 mL min^−1^ by using an HPLC pump (LaPrep P110, VWR International, Dublin, Ireland). The column experiments were conducted at 23 °C, the initial Cr(VI) concentration of the solution was 60 mg L^−1^, and the pH of the solution was two. Frequently, a sample was collected, and the concentration of Cr(VI) was determined.

## 4. Conclusions

Spruce sawdust is a low-cost and efficient absorbent for the removal of Cr(VI) from aqueous solutions. The maximum Cr(VI) adsorption capacity of organosolv-pretreated spruce sawdust using the Langmuir isotherm model is 318.3 mg g^−1^. The rate of adsorption was found to follow the pseudo-second-order kinetic model. It was observed that pretreatment with 50% diethylene glycol/50% H_2_O improves the adsorption capacity of sawdust by 84%, whereas pretreatment with 50% diethylene glycol/50% H_2_O/0.045 N H_2_SO_4_ enhances the adsorption capacity of sawdust by 89%.

According to adsorption kinetics, the material pretreated with organic solvent and acid catalyst (50% diethylene glycol/50% H_2_O/0.045 N H_2_SO_4_) at the optimal conditions, which were a temperature of 180 °C and pretreatment time of 50 min, adsorbed 3.7 times faster than the raw material (according to the intraparticle diffusion model).

Furthermore, our Thomas model showed that the material pretreated with organic solvent and acid catalyst (50% diethylene glycol/50% H_2_O/0.045 N H_2_SO_4_) at a temperature of 180 °C and an isothermal reaction time of 50 min has an adsorption capacity that is 13 times that of the untreated material. Overall, our results prove that the organosolv pretreatment of spruce sawdust produces a promising adsorbent candidate for the removal of Cr(VI).

Adsorption columns are widely used in water/wastewater treatment systems and their reliability makes these systems suitable for all types of facilities, e.g., large industrial-scale treatment plants with a high capacity. Furthermore, some pilot scale experiments are necessary to check the industrial reliability of the presented process.

## Figures and Tables

**Table 1:** Composition of the organosolv pretreated spruce sawdust.

No	Temperature *T_p_* (°C)	Time *t_p_* (min)	H_2_SO_4_ (N)	Cellulose (%)	Hemicelluloses (%)	Xylan (%)	Arabinan (%)	Mannan (%)	Lignin (%)	Other Components (%)	SRY (%)
1	Untreated			38.10	16.96	4.74	0.86	11.37	29.44	15.19	100
2	160	0	-	40.88	16.71	3.42	0.76	12.91	26.87	15.54	92.13
3	180	0	-	41.45	15.86	2.80	0.19	12.91	27.01	15.38	86.72
4	200	0	-	44.33	11.87	2.47	-	9.40	28.59	14.91	80.17
5	220	0	-	50.47	7.79	1.90	-	5.89	30.16	11.28	70.18
6	160	50	-	44.72	14.43	3.04	0.19	10.82	28.34	12.51	85.85
7	180	50	-	45.87	11.58	2.47	-	9.12	31.27	10.97	75.18
8	200	50	-	54.70	7.03	1.52	-	5.51	32.24	5.73	67.24
9	220	50	-	54.89	1.71	-	-	1.71	38.87	4.23	61.76
10	160	0	0.045	48.08	6.67	1.99	-	4.67	31.05	13.91	68.54
11	180	0	0.045	54.56	1.94	0.55	-	1.39	34.12	9.08	60.10
12	200	0	0.045	54.74	0.55	-	-	0.55	37.76	6.66	48.00
13	220	0	0.045	32.00	0.72	-	-	0.72	61.62	4.94	28.40
14	160	50	0.045	56.83	0.99	-	-	0.99	33.74	8.15	60.08
15	180	50	0.045	54.73	0.65	-	-	0.65	38.83	5.50	57.30
16	200	50	0.045	52.62	0.61	-	-	0.61	43.92	2.55	35.00
17	220	50	0.045	25.00	0.72	-	-	0.72	70.71	3.38	25.69

**Table 2:** BET surface area of the organosolv pretreated spruce sawdust.

Temperature *T_p_* (°C)	Time *t_p_* (min)	H_2_SO_4_ (N)	BET Surface Area (m^2^/g)
Untreated			0.703
160	0	-	0.812
180	0	-	1.567
200	0	-	2.600
220	0	-	2.849
160	50	-	2.893
180	50	-	2.594
200	50	-	2.968
220	50	-	4.078
160	0	0.045	0.517
180	0	0.045	2.323
200	0	0.045	8.613
220	0	0.045	9.392
160	50	0.045	1.248
180	50	0.045	2.026
200	50	0.045	8.700
220	50	0.045	11.335

**Table 3:** Freundlich isotherm model and Langmuir isotherm model parameters for Cr(VI) adsorption on untreated and organosolv pretreated spruce sawdust.

			Freundlich			Langmuir		
Temperature *T_p_* (°C)	Time *t_p_* (min)	H_2_SO_4_ (N)	*K_F_* [(mg g^−1^)(L mg^−1^)^1/n^]	*n*	SEE	*q_m_* (mg g^−1^)	*K_L_* (L mg^−1^*)*	SEE
Untreated			3.70	1.81	8.02	168.45	0.0043	6.16
160	0	-	4.80	2.14	6.44	110.68	0.0069	7.75
180	0	-	4.23	1.95	10.75	201.19	0.0022	11.84
200	0	-	7.48	2.32	3.97	120.95	0.0109	8.01
220	0	-	7.88	2.17	8.34	161.38	0.0089	5.78
160	50	-	6.21	2.26	9.38	113.64	0.0092	8.33
180	50	-	7.77	2.32	5.95	129.31	0.0101	7.08
200	50	-	4.78	1.70	8.17	265.31	0.0039	10.36
220	50	-	2.88	1.49	11.73	308.51	0.0027	10.30
160	0	0.045	5.97	2.09	6.18	142.83	0.0072	8.66
180	0	0.045	10.80	2.26	19.50	200.95	0.0082	19.62
200	0	0.045	14.25	2.31	7.78	225.20	0.0113	12.32
220	0	0.045	47.09	3.35	16.00	257.18	0.0705	20.82
160	50	0.045	6.21	1.88	8.10	216.42	0.0058	5.05
180	50	0.045	43.61	2.94	17.53	318.31	0.0333	18.01
200	50	0.045	19.11	2.81	17.58	170.35	0.0245	11.64
220	50	0.045	10.07	2.43	11.36	139.32	0.0143	6.82

**Table 4:** Estimated parameter values for the various isotherm models for Cr(VI) adsorption on untreated and organosolv pretreated spruce sawdust.

	*K_L_* (L mg^−1^)	*q_m_* (mg g^−1^)	*K_F_* [(mg g^−1^)(L mg^−1^)^1/n^]	*n*	SEE
**Untreated Spruce Sawdust**					
Freundlich			3.702	1.807	8.017
Langmuir	0.00426	168.45			6.157
Sips	0.00367	179.37		1.060	6.4541
Radle-Prausniz	0.00110	563.44		0.846	6.408
ModifiedRadle-Prausniz	0.00425	168.51		0.995	6.489
Toth	0.00430	168.00		1.0006	6.491
UNILAN	0.00425	168.45		−0.0016	6.489
**Pretreated Spruce Sawdust with 50% Diethylene Glycol, 50% Water, 220 °C, 50 min**					
Freundlich			2.884	1.494	11.725
Langmuir	0.00272	308.52			10.301
Sips	0.00272	308.22		1.003	10.857
Radle-Prausniz	0.00561	159.55		1.111	10.855
ModifiedRadle-Prausniz	0.00272	308.51		1.001	10.858
Toth	0.00293	311.04		1.013	10.856
UNILAN	0.00272	308.47		0.212	10.857
**Pretreated Spruce Sawdust with 50% Diethylene Glycol, 50% Water, 0.045 N Sulfuric Acid, 180 °C, 50 min**					
Freundlich			43.608	2.943	17.5321
Langmuir	0.0333	318.31			18.010
Sips	0.01161	436.48		1.651	10.8906
Radle-Prausniz	0.24487	101.65		1.242	14.175
ModifiedRadle-Prausniz	0.15610	131.80		1.274	14.848
Toth	0.47094	565.35		2.374	12.414
UNILAN	0.01251	432.44		3.238	13.880

**Table 5:** Cr(VI) adsorption capacity for various lignocellulosic materials.

	Freundlich		Langmuir				
Materials	*K_F_* [(mg g^−1^)(L mg^−1^)^1/n^]	*n*	*q_m_* (mg g^−1^)	*K_L_* (L mg^−1^)	pH	T (^o^C)	References
Magnetic biochar prepared from Melia azedarach wood	3.38	2.47	25.27	0.047	3	-	[41]
Mango kernel activated carbon	1.198	0.76	7.96	0.2634	2	35	[42]
Tea waste biomass	9.832	5621	33.33	0.0653	8	30	[43]
Wheat-residue black carbon	2.35	2.40	21.34	0.0288	1	30	[44]
Physically activated wood carbon	5.436	1.435	46.95	0.118	2	20	[45]
Physically activated date stone carbon	6.844	2.008	43.10	0.132		20	[45]
Virgin bamboo charcoal	1.524	1.258	7.58	0.013	2	25	[17]
Bamboo charcoal-based, cobalt-coated adsorbent	1.928	2.369	38.46	0.080	2	25	[17]
Cotton stalk peel	2.9	2.99	13.8	0.014	5.12	20	[46]
Cotton stalk peel (amine-cross linked)	0.36	3.74	117.9	0.024	5.12	20	[46]
Oak bark chars	0.523	2.016	4.619	0.073	2	25	[21]
Oak wood char	0.436	2.475	3.031	0.051	2	25	[21]
Pine sawdust (Autohydrolyzed)	8.928	4.776	345.9	0.00696	2	23	[28]
Spruce sawdust (diethylene glycol, water)	2.88	1.49	308.51	0.0027	2	23	In this work
Spruce sawdust (diethylene glycol, water, sulfuric acid)	43.61	2.94	318.31	0.0333	2	23	In this work

**Table 6:** Pseudo-first and pseudo-second order kinetic models parameters for Cr(VI) adsorption on untreated and organosolv pretreated spruce sawdust.

			Pseudo-First-Order Model			Pseudo-Second-Order Model		
Temperature *T_p_* (^o^C)	Time *t_p_* (min)	H_2_SO_4_ (N)	*k* (min^−1^)	*q* (mg g^−1^)	SEE	*k_2_* (g mg^−1^min^−1^)	*q* (mg g^−1^)	SEE
Untreated			0.0018	3.783	0.156	0.0003	5.225	0.133
160	0		0.0020	3.912	0.109	0.0003	5.438	0.100
180	0	-	0.0024	3.539	0.157	0.0005	4.686	0.131
200	0	-	0.0024	4.114	0.116	0.0004	5.400	0.079
220	0	-	0.0038	4.396	0.192	0.0007	5.376	0.139
160	50	-	0.0022	4.042	0.152	0.0003	5.419	0.136
180	50	-	0.0031	4.551	0.177	0.0005	5.768	0.144
200	50	-	0.0036	3.978	0.211	0.0007	4.914	0.139
220	50	-	0.0034	3.588	0.244	0.0008	4.443	0.181
160	0	0.045	0.0019	3.926	0.196	0.0003	5.376	0.175
180	0	0.045	0.0023	4.454	0.137	0.0003	5.987	0.106
200	0	0.045	0.0049	4.500	0.285	0.0010	5.318	0.178
220	0	0.045	0.0032	4.009	0.191	0.0006	5.096	0.141
160	50	0.045	0.0049	3.605	0.182	0.0013	4.273	0.109
180	50	0.045	0.0097	5.892	0.216	0.0021	6.486	0.150
200	50	0.045	0.0034	4.273	0.244	0.0006	5.321	0.163
220	50	0.045	0.0021	4.188	0.149	0.0003	5.713	0.126

**Table 7:** Inta-particle diffusion kinetic model parameters for Cr(VI) adsorption on untreated and organosolv pretreated spruce sawdust.

			Intra-Particle Diffusion Model		
Temperature *T_p_* (^o^C)	Time *t_p_* (min)	H_2_SO_4_ (N)	*k_p_* (mg g^−1^min^−1/2^)	*c* (mg g^−1^)	SEE
Untreated			0.1021	−0.1831	0.091
160	0		0.1133	−0.281	0.160
180	0	-	0.1011	−0.071	0.128
200	0	-	0.1203	−0.141	0.147
220	0	-	0.1655	−0.128	0.163
160	50	-	0.1147	−0.146	0.151
180	50	-	0.1541	−0.155	0.185
200	50	-	0.1527	−0.182	0.166
220	50	-	0.1198	−0.002	0.190
160	0	0.045	0.1069	−0.142	0.144
180	0	0.045	0.1399	−0.324	0.149
200	0	0.045	0.1721	−0.148	0.203
220	0	0.045	0.1563	−0.356	0.200
160	50	0.045	0.1662	−0.150	0.122
180	50	0.045	0.3768	−0.180	0.139
200	50	0.045	0.1837	−0.388	0.166
220	50	0.045	0.1233	−0.287	0.178

**Table 8:** Bohart-Adams, Thomas, and Modified Dose-Response column models parameters for Cr(VI) adsorption on untreated and organosolv pretreated spruce sawdust.

	Untreated	160 °C, 50 min	180 °C, 50 min	200 °C, 50 min	220 °C, 50 min
**Bohart-Adams Model**					
*N* (mg L^−1^)	662	1154	7814	1665	998
*K* (L mg^−1^ min^−1^)	0.00012	0.000033	0.000016	0.000050	0.000053
SEE	3.593	4.530	1.123	2.947	4.063
**Thomas Model**					
*q*_0_ (mg g^−1^)	2.437	5.148	31.08	7.425	4.452
*k_T_* (L mg^−1^ min^−1^)	0.00012	0.000033	0.000016	0.000050	0.000053
SEE	3.593	4.530	1.123	2.947	4.063
**Modified Dose-Response Model**					
*q*_0_ (mg g^−1^)	2.222	4.377	30.13	6.895	3.988
*a_mdr_*	2.484	1.425	4.029	2.315	1.487
SEE	1.976	3.138	1.682	2.661	2.942

**Table 9:** Comparison with other adsorbents/biosorbents in a column study.

Materials	*k_T_* (L mg^−1^ min^−1^)	*q*_0_ (mg g^−1^)	Reference
Spirulina platensis	0.00178	6.087	[56]
Strychnos nux vomica tree fruit shell	0.00018	101.8	[57]
Auto-hydrolyzed pine sawdust	0.00024	18.87	[58]
Modified corn stalk	0.00095	152.3	[59]
Nanosorbents from magnetite Impregnated Phaseolus vulgaris husk	0.00018	53.01	[60]
Spruce sawdust (diethylene glycol, water, sulfuric acid)	0.000016	31.08	In this work

**Table 10:** FTIR peaks for untreated and pretreated spruce sawdust.

Wavenumber [cm^−1^]			Assignment	Components
Untreated	Pretreated	Differences		
3464	3347	117	O-H stretching	Cellulose, Hemicelluloses, Lignin
2909	2942	−33	C-H stretching	Cellulose, Hemicelluloses, Lignin
2362	2364	−2	N-H stretching	Cellulose, Hemicelluloses, Lignin
1735	1700	35	C=O stretching	Hemicelluloses, Lignin
1654	1653	1	Aromatic skeletal vibration, C=O stretching, adsorbed O-H	Hemicelluloses, Lignin
-	1616	-	C=C stretching of phenol group	Cellulose, Hemicelluloses, Lignin
1507	1509	−2	C=C-C aromatic ring stretching and vibration	Lignin
1435	1457	−22	C-H deformation (in methyl and methylene)	Lignin
1374	1376	−2	C-H bending, C-H stretching in CH3	Cellulose, Hemicelluloses, Lignin
1335	1320	15	CH2 wagging, C-O stretching of C5 substituted aromatic units	Cellulose, Hemicelluloses, Lignin
1268	1281	−13	C-O stretching of guaiacyl unit	Lignin
1134	1132	2	C-O-C stretching	Cellulose, Hemicelluloses
1043	1068	−25	C-OH stretching vibration, C-O deformation	Cellulose, Hemicelluloses, Lignin
-	1039	-	C-O stretching, aromatic C-H in plane deformation	Cellulose, Lignin
902	855	47	C-O-C stretching	Cellulose, Hemicelluloses
805	851	−46	Aromatic C-H out of plane bending	Lignin

**Table 11:** FTIR peaks for spruce sawdust: before and after Cr(VI) adsorption on pretreated material.

Wavenumber [cm^−1^]			Assignment	Components
Untreated	Pretreated	Differences		
3347	3410	−63	O-H stretching	Cellulose, Hemicelluloses, Lignin
2942	2939	3	C-H stretching	Cellulose, Hemicelluloses, Lignin
2364	2365	−1	N-H stretching	Cellulose, Hemicelluloses, Lignin
1700	1684	16	C=O stretching	Hemicelluloses, Lignin
1653	1651	2	Aromatic skeletal vibration, C=O stretching, adsorbed O-H	Hemicelluloses, Lignin
1616	1617	−1	C=C stretching of phenol group	Cellulose, Hemicelluloses, Lignin
1509	1508	1	C=C-C aromatic ring stretching and vibration	Lignin
1457	1455	2	C-H deformation (in methyl and methylene)	Lignin
1376	1375	1	C-H bending, C-H stretching in CH3	Cellulose, Hemicelluloses, Lignin
1320	1314	6	CH2 wagging, C-O stretching of C5 substituted aromatic units	Cellulose, Hemicelluloses, Lignin
1281	1283	−2	C-O stretching of guaiacyl unit	Lignin
1132	1133	−1	C-O-C stretching	Cellulose, Hemicelluloses
1068	1076	−8	C-OH stretching vibration, C-O deformation	Cellulose, Hemicelluloses, Lignin
1039	1038	1	C-O stretching, aromatic C-H in plane deformation	Cellulose, Lignin
855	899	−44	C-O-C stretching	Cellulose, Hemicelluloses
851	850	1	Aromatic C-H out of the plane bending	Lignin

## References

[1] Ali H., Khan E., Ilahi I. (2019). Environmental Chemistry and Ecotoxicology of Hazardous Heavy Metals: Environmental Persistence, Toxicity, and Bioaccumulation. J. Chem. N. Y..

[2] Junaid M., Hashmi M.Z., Malik R.N., Pei D.-S. (2016). Toxicity and oxidative stress induced by chromium in workers exposed from different occupational settings around the globe: A review. Environ. Sci. Pollut. Res. Int..

[3] Mojdeh O., Mohamed K.A., Wan A.W.D., Saeid B. (2009). Removal of Hexavalent Chromium-Contaminated Water and Wastewater: A Review. Water Air Soil Pollut..

[4] Ahmed M.F., Mokhtar M.B. (2020). Assessing Cadmium and Chromium Concentrations in Drinking Water to Predict Health Risk in Malaysia. Int. J. Environ. Res. Public Health.

[5] Economou-Eliopoulos M., Megremi I., Vasilatos C. (2011). Factors controlling the heterogeneous distribution of Cr(VI) in soil, plants and groundwater: Evidence from the Assopos basin, Greece. Geochemistry.

[6] Oliveira H. (2012). Chromium as an Environmental Pollutant: Insights on Induced Plant Toxicity. J. Bot..

[7] Khulbe K.C., Matsuura T. (2018). Removal of heavy metals and pollutants by membrane adsorption techniques. Appl. Water Sci..

[8] Barakat M.A. (2011). New trends in removing heavy metals from industrial wastewater. Arab. J. Chem..

[9] Crini G., Lichtfouse E. (2018). Wastewater treatment: An overview. Book Green Adsorbents for Pollutant Removal.

[10] Crini G., Lichtfouse E., Wilson L.D., Morin-Crini N. (2018). Adsorption-Oriented Processes Using Conventional and Non-conventional Adsorbents for Wastewater Treatment. Book Green Adsorbents for Pollutant Removal.

[11] Namasivayam C., Sureshkumar M.V. (2008). Removal of chromium(VI) from water and wastewater using surfactant modified coconut coir pith as a biosorbent. Bioresour. Technol..

[12] Tsamo C., Bachirou I., Samomssa I., Fouogoung T.B. (2019). Removal of Hexavalent Chromium from Aqueous Solution Using Unmodified SawDust: Batch and Column Studies. Curr. J. Appl..

[13] Moussavi G., Barikbin B. (2010). Biosorption of chromium(VI) from industrial wastewater onto pistachio hull waste biomass. Chem. Eng. J..

[14] Vaghetti J.C.P., Lima E.C., Royer B., Brasil J.L., Da Cunha B.M., Simon N.M., Cardoso N.F., Zapata-Norena C.P. (2008). Application of Brazilian-pine fruit coat as a biosorbent to removal of Cr(VI) from aqueous solution -Kinetics and equilibrium study. Biochem. Eng. J..

[15] Tejada-Tovar C., Herrera-Barros A., Villabona-Ortíz A. (2020). Assessment of Chemically Modified Lignocellulose Waste for the Adsorption of Cr(VI). Rev. Fac. Ing..

[16] Saranya K., Palanisami T., Mallavarapu M., Kadiyala V., Yong B.L., Ravi N. (2016). Potential of *Melaleuca diosmifolia* leaf as a low-cost adsorbent for hexavalent chromium removal from contaminated water bodies. Process Saf. Environ. Protect..

[17] Wang Y., Wang X.J., Liu M., Wang X., Wu Z., Yang L.Z., Xia S.Q., Zhao J.F. (2012). Cr(VI) removal from water using cobalt-coated bamboo charcoal prepared with microwave heating. Ind. Crop. Prod..

[18] Altun T., Pehlivan E. (2012). Removal of Cr(VI) from aqueous solutions by modified walnut shells. Food Chem..

[19] Pourfadaraki S., Jorfi S., Ahmadi M., Takdastan A. (2017). Experimental data on adsorption of Cr(VI) from aqueous solution using nanosized cellulose fibers obtained from rice husk. Data Brief.

[20] Shi C., Qiao Y., An X., Tian Y., Zhou H. (2020). High-capacity adsorption of Cr(VI) by lignin-based composite: Characterization, performance and mechanism. Int. J. Biol. Macromol..

[21] Mohan D., Rajput S., Singh V.K., Steele P.H., Pittman C.U. (2011). Modeling and evaluation of chromium remediation from water using low cost bio-char, a green adsorbent. J. Hazard. Mater..

[22] Garg V.K., Gupta R., Kumar R., Gupta R.K. (2004). Adsorption of chromium from aqueous solution on treated sawdust. Bioresour. Technol..

[23] Lin C., Luo W., Luo T., Zhou Q., Li H., Jing L. (2018). A study on adsorption of Cr(VI) by modified rice straw: Characteristics, performances and mechanism. J. Clean. Prod..

[24] Vilardi G., Ochando-Pulido J.M., Verdone N., Stoller M., Di Palma L. (2018). On the removal of hexavalent chromium by olive stones coated by iron-based nanoparticles: Equilibrium study and chromium recovery. J. Clean. Prod..

[25] Mohamed H.I., Naimah I., Hamidi A.A., Mohd N.A., Nor Habsah Md.S., Ali A.L.Z., Shamsul R.M.K. (2008). Removal of chromium (VI) from aqueous solution using treated oil palm fibre. J. Hazard. Mater..

[26] Yang J., Yu M., Chen W. (2015). Adsorption of hexavalent chromium from aqueous solution by activated carbon prepared from longan seed: Kinetics, equilibrium and thermodynamics. J. Ind. Eng. Chem..

[27] Wang B., Sun Y.-C., Sun R.-C. (2019). Fractionational and structural characterization of lignin and its modification as biosorbents for efficient removal of chromium from wastewater: A review. J. Leather Sci. Eng..

[28] Sidiras D., Politi D., Batzias F., Boukos N. (2013). Efficient removal of hexavalent chromium from aqueous solutions using autohydrolyzed Scots Pine (Pinus Sylvestris) sawdust as adsorbent. Int. J. Environ. Sci. Technol..

[29] Sidiras D., Batzias F., Ranjan R., Tsapatsis M. (2011). Simulation and optimization of batch autohydrolysis of wheat straw to monosaccharides and oligosaccharides. Bioresour. Technol..

[30] Park Y.C., Kim J.S. (2012). Comparison of various alkaline pretreatment methods of lignocellulosic biomass. Energy.

[31] Batzias F., Sidiras D., Schroeder E., Weber C. (2009). Simulation of dye adsorption on hydrolyzed wheat straw in batch and fixed-bed systems. Chem. Eng. J..

[32] Maarten A., Kootstra J., Beeftink H.H., Scott E.L., Sanders J.P.M. (2009). Comparison of dilute mineral and organic acid pretreatment for enzymatic hydrolysis of wheat straw. Biochem. Eng. J..

[33] Zhang K., Pei Z., Wang D. (2016). Organic solvent pretreatment of lignocellulosic biomass for biofuels and biochemicals: A review. Bioresour. Techno..

[34] Salapa I., Topakas E., Sidiras D. (2018). Simulation and optimization of barley straw organosolv pretreatment. Ind. Crops Prod..

[35] Freundlich H.M.F. (1906). Über die adsorption in lösungen, Zeitschrift für Physikalische Chemie. Phys. Chem..

[36] Langmuir I. (1916). The constitution and fundamental properties of solids and liquids. J. Am. Chem. Soc..

[37] Sips R. (1948). Structure of a catalyst surface. J. Chem. Phys..

[38] Radke C.J., Prausnitz J.M. (1972). Adsorption of Organic Solutes from Dilute Aqueous Solution on Activated Carbon. Ind. Eng. Chem. Fundam..

[39] Chern J.M., Wu C.Y. (2001). Desorption of dye from activated carbon beds: Effects of temperature, pH, and alcohol. Wat. Res..

[40] Toth J. (2000). Calculation of the BET-compatible surface area from any Type I isotherms measured above the critical temperature. J. Colloid Interface Sci..

[41] Zhang X., Lv L., Qin Y., Xu M., Jia X., Chen Z. (2018). Removal of aqueous Cr(VI) by a magnetic biochar derived from Melia azedarach wood. Bioresour. Technol..

[42] Rai M.K., Shahi G., Meena V., Meena R., Chakraborty S., Singh R.S., Rai B.N. (2016). Removal of hexavalent chromium Cr(VI) using activated carbon prepared from mango kernel activated with H_3_PO_4_. Resour. Effic. Technol..

[43] Gupta A., Balomajumder C. (2015). Simultaneous adsorption of Cr(VI) and phenol onto tea waste biomass from binary mixture: Multicomponent adsorption, thermodynamic and kinetic study. J. Environ. Chem. Eng..

[44] Wang X.S., Chen L.F., Li F.Y., Chen K.L., Wan W.Y., Tang Y.J. (2010). Removal of Cr(VI) with wheat-residue derived black carbon: Reaction mechanism and adsorption performance. J. Hazard. Mater..

[45] Danish M., Hashim R., Ibrahim M.N.M., Rafatullah M., Sulaiman O. (2012). Surface characterization and comparative adsorption properties of Cr(VI) on pyrolysed adsorbents of Acacia mangium wood and Phoenix dactylifera L. stone carbon. J. Anal. Appl. Pyrolysis..

[46] Xu X., Gao B.-Y., Tang X., Yue Q.-Y., Zhong Q.-Q., Li Q. (2011). Characteristics of cellulosic amine-crosslinked copolymer and its sorption properties for Cr(VI) from aqueous solutions. J. Hazard. Mater..

[47] Lagergren S. (1898). Zur theorie der sogenannten adsorption gelöster stoffe. Kungliga Svenska Vetenskapsakademiens. Handlingar.

[48] Ho Y.S., Ng J.C.Y., McKay G. (2000). Kinetics of pollutants sorption by biosorbents: Review. Sep. Purif. Methods..

[49] Weber W.J., Morris J.C. (1963). Kinetics of adsorption on carbon from solution. J. Sanit. Eng. Div. Am. Soc. Civ. Eng..

[50] Norouzi S., Heidari M., Alipour V., Rahmanian O., Fazlzadeh M., Mohammadi-moghadam F., Nourmoradi H., Goudarzi B., Dindarloo K. (2018). Preparation, characterization and Cr(VI) adsorption evaluation of NaOH—activated carbon produced from Date Press Cake; an agro-industrial waste. Bioresour. Technol..

[51] Bouaziz F., Koubaa M., Kallel F., Chaari F., Driss D., Ghorbel R.E., Chaabouni S.E. (2015). Efficiency of almond gum as a low-cost adsorbent for methylene blue dye removal from aqueous solutions. Ind. Crops Prod..

[52] Bohart G., Adams E.N. (1920). Some aspects of the behavior of charcoal with respect to chlorine. J. Am. Chem. Soc..

[53] Thomas H.C. (1944). Heterogeneous ion exchange in a flowing system. J. Am. Chem. Soc..

[54] Chu K.H. (2010). Fixed bed sorption: Setting the record straight on the Bohart–Adams and Thomas models. J. Hazard. Mater..

[55] Yan G., Viraraghavan T., Chen M. (2001). A new model for heavy metal removal in a biosorption column. Adsorpt. Sci. Technol..

[56] Gokhale S.V., Jyoti K.K., Lele S.S. (2009). Modeling of chromium(VI) biosorption by immobilized Spirulina platensis in packed column. J. Hazard. Mater..

[57] Nakkeeran E., Patra C., Shahnaz T., Rangabhashiyam S., Selvaraju N. (2018). Continuous biosorption assessment for the removal of hexavalent chromium from aqueous solutions using Strychnos nux vomica fruit shell. Bioresour. Technol. Rep..

[58] Sidiras D., Politi D. Simulation and optimization of hexavalent chromium adsorption on autohydrolyzed Scots Pine (Pinus Sylvestris) sawdust in batch and fixed-bed systems. Proceedings of the Second International Conference on Advances in Bio-Informatics, Bio-Technology and Environmental Engineering-ABBE.

[59] Chen S., Yue Q., Gao B., Li Q., Xu X., Fu K. (2012). Adsorption of hexavalent chromium from aqueous solution by modified corn stalk: A fixed-bed column study. Bioresour. Technol..

[60] Srivastava S., Agrawal S.B., Mondal M.K. (2018). Fixed Bed Column Adsorption of Cr(VI) from Aqueous Solution Using Nanosorbents Derived from Magnetite Impregnated Phaseolus vulgaris Husk. Environ. Prog. Sustain. Energy.

[61] Chen C., Zhao P., Li Z., Tong Z. (2016). Adsorption behavior of chromium(VI) on activated carbon from eucalyptus sawdust prepared by microwave-assisted activation with ZnCl_2_. Desalin. Water Treat..

[62] Gupta S., Babu B.V. (2009). Removal of toxic metal Cr(VI) from aqueous solutions using sawdust as adsorbent: Equilibrium, kinetics and regeneration studies. Chem. Eng. J..

[63] Pan S.Y., Syu W.J., Chang T.K., Lee C.H. (2020). A multiple model approach for evaluating the performance of time-lapse capsules in trapping heavy metals from water bodies. RSC Adv..

[64] Karthikeyan T., Rajgopal S., Miranda L.R. (2005). Chromium(VI) adsorption from aqueous solution by Hevea Brasilinesis sawdust activated carbon. J. Hazard. Mater..

[65] Albadarina A.B., Al-Muhtasebb A.H., Al-laqtaha N.A., Walker G.M., Allena S.J., Ahmada M.N.M. (2011). Biosorption of toxic chromium from aqueous phase by lignin: Mechanism, effect of other metal ions and salts. Chem. Eng. J..

[66] Gurgel L.V., Perin de Melo J.C., de Lena J.C., Gil L.F. (2009). Adsorption of chromium (VI) ion from aqueous solution by succinylated mercerized cellulose functionalized with quaternary ammonium groups. Bioresour. Technol..

[67] Demirbaş A. (2005). Adsorption of Cr(III) and Cr(VI) Ions from Aqueous Solutions on to Modified Lignin. Energy Sources.

[68] Tazrouti N., Amrani M. (2009). Chromium (Vi) Adsorption onto Activated Kraft Lignin Produced from Alfa Grass (Stipa Tenacissima). Bioresources.

[69] Zhuang J., Li M., Pu Y., Ragauskas A.J., Yoo C.G. (2020). Observation of Potential Contaminants in Processed Biomass Using Fourier Transform Infrared Spectroscopy. Appl. Sci..

[70] Ahmad A., Rafatullah M., Sulaiman O., Ibrahim M.H., Hashim R. (2009). Scavenging behaviour of meranti sawdust in the removal of methylene blue from aqueous solution. J. Hazard. Mater..

[71] Rafatullah M., Sulaiman O., Hashim R., Ahmad A. (2012). Removal of cadmium (II) from aqueous solutions by adsorption using meranti wood. Wood. Sci. Technol..

[72] González-Peña M.M., Hale M.D.C. (2011). Rapid assessment of physical properties and chemical composition of thermally modified wood by mid-infrared spectroscopy. Wood Sci. Technol..

[73] Fackler K., Stevanic J.S., Ters T., Hinterstoisser B., Schwanninger M., Salmen L. (2010). Localisation and characterisation of incipient brown-rot decay within spruce wood cell walls using FT-IR imaging microscopy. Enzym. Microb. Technol..

[74] Saeman J.F., Bubl J.F., Harris E.E. (1945). Quantitative saccharification of wood and cellulose. Ind. Eng. Chem. Anal. Ed..

[75] Salapa I., Katsimpouras C., Topakas E., Sidiras D. (2017). Organosolv pretreatment of wheat straw for efficient ethanol production using various solvents. Biomass Bioenergy.

[76] Tappi Standards (1997). Tappi Tests Methods, T222 om-88 Atlanta. https://www.tappi.org/content/SARG/T222.pdf.

[77] (1975). DIN 66132: Determination of Specific Surface Area of Solids by Adsorption of Nitrogen; Single Point Differential Method According to Haul and Dümbgen.

